# Hepatitis B Core Antigen in Hepatocytes of Chronic Hepatitis B: Comparison between Indirect Immunofluorescence and Immunoperoxidase Method

**DOI:** 10.5005/jp-journals-10018-1120

**Published:** 2015-01-06

**Authors:** ,Ruksana Raihan, Shahina Tabassum, Mamun Al-Mahtab, Afzalun Nessa, Munira Jahan, Chowdhury Mohammad Shamim Kabir, Mohammad Kamal, Julio Cesar Aguilar

**Affiliations:** 1 Department of Microbiology, Faculty of Medicine, AIMST University, Bedong, Kedah, Malaysia; 2 Department of Virology, Bangabandhu Sheikh Mujib Medical University, Shahbag, Dhaka, Bangladesh; 3 Department of Hepatology, Bangabandhu Sheikh Mujib Medical University, Shahbag, Dhaka, Bangladesh; 4 Department of Medicine, Shaheed Suhrawardy Medical College and Hospital, Mirpur, Dhaka, Bangladesh; 5 Department of Pathology, Bangabandhu Sheikh Mujib Medical University, Shahbag, Dhaka, Bangladesh; 6 Department of Biomedical Research, Centre for Genetic Engineering and Biotechnology, Havana City, Cuba

**Keywords:** Chronic hepatitis B infection, Hepatitis B core antigen, Indirect immunofluorescence, Indirect immunoperoxidase.

## Abstract

**Background:**

Hepatitis B virus (HBV) infection has many faces. Precore and core promoter mutants resemble inactive carrier status. The identification of hepatitis B core antigen (HBcAg) in hepatocytes may have variable clinical significance. The present study was undertaken to detect HBcAg in chronic hepatitis B (CHB) patients and to assess the efficacy of detection system by indirect immunofluorescence (IIF) and indirect immunoperoxidase (IIP).

**Materials and methods:**

The study was done in 70 chronic HBV-infected patients. Out of 70 patients, eight (11.4%) were hepatitis B e antigen (HBeAg) positive and 62 (88.57%) were HBeAg negative. Hepatitis B core antigen was detected by indirect immunofluorescence (IIF) and indirect immunoperoxidase (IIP) methods in liver tissue.

**Results:**

All HBeAg positive patients expressed HBcAg by both IIF and IIP methods. Out of 62 patients with HBeAg-negative CHB, HBcAg was detected by IIF in 55 (88.7%) patients and by IIP in 51 (82.26%) patients. A positive relation among viral load and HBcAg detection was also found. This was more evident in the case of HBeAg negative patients and showed a positive relation with HBV DNA levels.

**Conclusion:**

Hepatitis B core antigen can be detected using the IIF from formalin fixed paraffin block preparation and also by IIP method. This seems to reflect the magnitudes of HBV replication in CHB.

**How to cite this article:**

Raihan R, Tabassum S, Al-Mahtab M, Nessa A, Jahan M, Kabir CMS, Kamal M, Aguilar JC. Hepatitis B Core Antigen in Hepatocytes of Chronic Hepatitis B: Comparison between Indirect Immunofluorescence and Immunoperoxidase Method. Euroasian J Hepato-Gastroenterol 2015;5(1):7-10.

## INTRODUCTION

Globally, hepatitis B virus (HBV) is one of the most common infectious diseases and the major cause of chronic hepatitis, cirrhosis, and hepatocellular carcinoma (HCC).^1-4^ Bangladesh is a densely populated country with intermediate endemicity (2 to 7%) for chronic hepatitis B (CHB), where the lifetime risk of acquiring HBV infection is between up to 60%.^5^ Various studies from Bangladesh have shown that HBV is responsible for 31.25% cases of acute hepatitis, 76.3% cases of chronic hepatitis, 61.15% cases of cirrhosis of liver and 33.3% cases of HCC.^6-8^

Hepatits B virus is a 42 nm particle. The major antigens comprising HBV are the hepatitis B surface antigen (HBsAg) and the hepatitis B core antigen (HBcAg). A secreted variant of the nucleocapside antigen, the hepatitis B e antigen (HBeAg) can be detected in the blood. However, HBcAg is only detectable in the hepatocytes, both in nucleus or cytoplasm.^9^ Presence of HBcAg in hepatocyte is related to the presence of HBeAg as a marker of HBV replication. However, a significant proportion of patients with CHB are infected with the mutant HBV which decreases or abolishes the production of HBeAg due to mutation of precore or core promoter region.^10,11^ Such variants, called HBeAg negative CHB, they are commonly found in Mediterranean and Asian countries.^10^ Detection of circulating HBV DNA and HBcAg in liver tissues may be an indicator of active viral replication compared to HBeAg.^11,12^ Both indirect immunofluorescence (IIF) and indirect immunoperoxidase (IIP) can be used to detect HBcAg in hepatocytes. In the present study, we applied both methods for the detection of HBcAg in hepatocytes and compared their expressions in formalin fixed and paraffin-embedded liver tissue of CHB patients.

## MATERIALS AND METHODS

### Study Design

This cross-sectional study was carried out among 70 patients who were incidentally diagnosed as CHB patients and underwent liver biopsy as part of their routine clinical management.

### Subjects

Patients were selected from the Inpatient Department of Hepatology, Bangabandhu Sheikh Mujib Medical University (BSMMU) Hospital, Dhaka, Bangladesh, and laboratory works were performed at the Department of Virology of BSMMU. Patients were expressing HBsAg for at least 6 months with serum HBeAg positive or negative and had detectable serum HBV DNA. Patients gave written consent for the study. Patients with the history of significant alcohol consumption (> 20 gm/day), renal disease, heart disease or malignancy, and with detectable antibodies to human immunodeficiency virus (HIV) and hepatitis C virus (HCV), history of previous antiviral treatment were excluded from the study.

### Sample Collection

Under all aseptic precautions, a trucut liver biopsy was done by a hepatologist. The tissues were fixed in formalin and embedded in paraffin for routine histological study and for IIF staining and indirect IIP staining.

### Staining Procedure

Five micrometer thick tissue sections from paraffin blocks were taken on albumin coated slides and incubated at 37°C overnight for 16 hours. Then, after deparaffiniza-tion intrahepatic expression of HBcAg were studied by using polyclonal rabbit anti-HBcAg (Dako, Carpinteria, CA, USA) as primary antibody and the color reaction was enhanced by Envision Detection Kit (Dako) from formalin fixed paraffin embedded tissues for immuno-peroxidase method. Immunofluorescence staining was done by fluorescein isothiocyanate conjugated secondary antibody (Polyclonal Swine Anti-Rabbit Immunoglo-bulin/FITC, Dako). The amount of HBcAg in liver was also semiquantitatively scored according to the proportion of hepatocytes that stained positive on a 0 to 3+ scale (0%—absent, 1 to 10% —grade 1, 11 to 50%—grade 2, > 50%—grade 3).^13^ Intra-cellular localization of HBcAg was labeled as nuclear, cytoplasmic or mixed type (mixed but predominantly nuclear and mixed but predominantly cytoplasmic).

### Other Methods

Virological (HBV DNA) and serological (serum HBsAg and HBeAg) tests were conducted at Labaid Hospital, Dhaka, by conventional methods.

## STATISTICAL ANALYSIS

Statistical analysis was done by Prism Software, version 4. Results were expressed as percentage, mean and standard deviation. For comparison between two methods, Kappa statistic of agreement test and validity test were done.

## RESULTS

The present study was carried out among 70 serologically diagnosed CHB patients. Out of total 70 patients, 58 were males and 12 were females with a mean age of 30.51 years.

All eight patients (11.43%) that were HBeAg positive expressed HBcAg in the liver (Table 1). However, 55 of 62 patients HBeAg negative cases expressed HBcAg by IIF method and 51 of 62 patients showed HBcAg in the liver by IIP method.

**Table 1:** **Table 1:** Relation of HBcAg and HBV DNA according to HBeAg status of patients

		*DNA*		*HBcAg by IIF*					
*HBeAg*		*(copies/ml)*		*Positive*		*Negative*		*Total*	
Positive (n = 8)		≥10^5^		8 (100.0)#		—		8 (100.0)	
Negative (n = 55)		<10^5^		37 (67.3)		5(71.4)		42 (67.7)	
		≥10^5^		18 (32.7)		2 (28.6)		20 (32.3)	

#Figure within parentheses indicates in percentage

The HBV DNA profile of the study patients were categorized into high (≥10^5^ copies/ml) and low (< 10^5^ copies/ ml). All eight (100%) patients who were HBeAg positive had high levels of HBV DNA. Among HBeAg negative and HBcAg-expressing patients, 37 (67.3%) had low HBV DNA (< 10^5^ copies/ml), while 18 (32.7°%) patients had high circulating HBV DNA (≥10^5^ copies/ml). Among the seven cases with HBeAg-positive and HBcAg-negative, five (71.4%) had low HBV DNA and two (28.6%) had high HBV DNA (Table 1). Most (7 out of 8) HBeAg positive patients had grade 3 HBcAg with a mean HBV DNA of 1.2 × 10^11^ ± 3.3 × 10^11^ copies/ml. The patient classified as grade 1 had a comparatively low HBV DNA load (6.4 × 10^6^ copies/ml).

**Graph 1:**
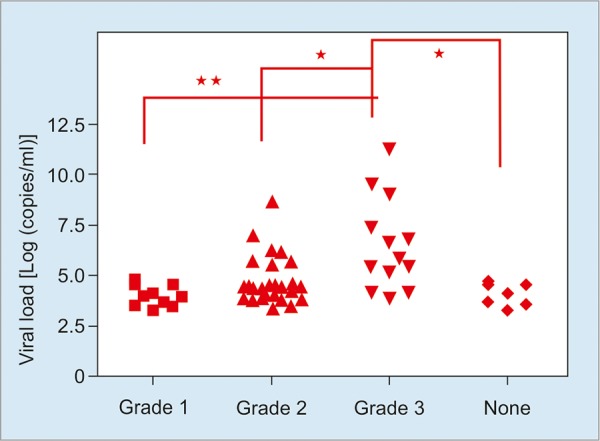
Relation between the HBV DNA load and the HBcAg grade among HBeAg negative patients (**p < 0.01; *p < 0.05)

The levels of HBV DNA in patients with grades 1, 2 and 3 expression of HBcAg in the liver were 1.6 × 10^4^ ± 1.88 × 10^4^ copies/ml, 1.36 × 10^7^ ± 7.35 × 10^7^ copies/ml, 1.6 × 10^10^ ± 5.87 × 10^10^ copies/ml respectively. There was no statistical differences among grades 1, 2 and none groups but there was a significant difference in grade 3 and 2, grade 3 and none (p < 0.05), between grade 3 and 1 (p < 0.01) (Graph 1).

Out of 70 cases, 59 were positive for HBcAg by both IIP and indirect IIF methods. Eleven cases were negative for core antigen by IIP method, among which seven were negative by both the methods, but four of this 11 were positive in IIF methods. Validity test showed that sensitivity of IIF was 100%, specificity was 63.6% and accuracy was 94.3%.

## DISCUSSION

The detection of HBcAg in liver biopsy samples from Bangladeshi CHB patients explored the antigen expression and their relation to virological variables using two staining methods. Previous studies showed that HBeAg positive CHB patients have higher serum HBV DNA levels than HBeAg negative patients.^14-16^ All HBeAg positive cases were positive for HBcAg in hepatocytes. This result was similar with a study from Taiwan which found 100% of HBcAg expression in hepatocytes among HBeAg positive patients.^17^ A study conducted in Korea also observed high prevalence (92%) in this group of patients.^13^

Interestingly, among HBeAg negative cases, a very high percentage of patients (88.7%) were HBcAg positive in hepatocytes compared to reporting from Asia, were the HBcAg detection was 59% in Korea.^13,17^ It would be important to examine in the future the influence of regional HBV genotypes in the HBcAg hepatocytes expression. Present results were further confirmed by IIF in more than 90% of the samples to avoid any technique related variability.^18^

Only with one exception, all HBeAg positive patients were classified as grade 3 according to HBcAg level in hepatocytes. Hence, it was not possible to establish a relation between viral load and HBcAg grade. However, for HBeAg negative cases, it was found a more homogeneous distribution among grades. In such patients, the increase in the grade of HBcAg detection in hepatocytes was related to the increase of HBV DNA, suggesting that grading HBcAg expression in hepatocytes may be a useful marker for replication in case of HBeAg negative patients. Thus, liver biopsy results could be complemented with further virological information, increasing the value of this aggressive procedure. The detection of HBcAg in hepatocytes has been considered a marker of active replication.^19^

The finding of disease progression in patients with low-viral load among patients with HBeAg negative serology in Bangladesh remains a matter of concern.^20^ Other countries have referred similar results.^21,22^ This particular variable—HBcAg grade—could provide important information in these patients as serum HBV DNA fluctuates in HBeAg negative patients and the decision to start treatment is a complex issue now-adays.^23,24^ Both IIF and IIP tests were performed in the present study to detect HBV core antigen. According to the performance and agreement tests, IIF was found to be an alternative to the IIP method. Although IIP method is a well-accepted and well-practiced method throughout the world, it is costly and needs very skilled hands from the technical point of view. In contrast, IIF method is cost-effective, less time consuming and more user-friendly. Therefore, IIF maybe used as an alternative to IIP method.
